# Satisfaction with the quality of nursing care among older adults during acute hospitalization in Ghana

**DOI:** 10.1002/nop2.1169

**Published:** 2022-01-05

**Authors:** Abdul‐Ganiyu Fuseini, Rahinatu Bayi, Afizu Alhassan, Joseph Aniba Atomlana

**Affiliations:** ^1^ School of Nursing and Midwifery Deakin University Melbourne Australia; ^2^ Zebilla District Hospital Upper East Region Zebilla Ghana; ^3^ Nursing and Midwifery Training College, Kpembe Salaga Ghana; ^4^ Savelugu RCH center Ghana health Service Savelugu Ghana

**Keywords:** care, health, nursing, older adults, quality, satisfaction

## Abstract

**Aim:**

This study assessed the level of satisfaction with the quality of nursing care among hospitalized older adults and the factors associated with it.

**Design:**

This was a quantitative descriptive cross‐sectional survey.

**Methods:**

We used a validated questionnaire to collect data from a convenience sample of 206 older adults from three government hospitals in Tamale, Ghana. Data were analysed using descriptive statistics, independent sample *t* test and one‐way ANOVA.

**Results:**

Most of the participants (72.3%) reported moderate levels of satisfaction with the quality of nursing care, while 23.8% reported high levels of satisfaction. The association between gender, religion and level of satisfaction with nursing care was not statistically significant. However, patients differed on levels of satisfaction based on healthcare facility: patients at the Tamale Central Hospital were more satisfied with the quality of nursing care than those at the Tamale Teaching Hospital. There is a need for capacity building and sensitization workshops on the rudiments of geriatric care for nurses in the metropolis to serve as an impetus for improvement in the quality of care.

## 1 INTRODUCTION

The increase in global life expectancy from 67 years to 72 years between 2000 and 2016 has resulted in a growing proportion of older adults across the globe (Ailshire & Crimmins, 2011; Cho et al., 2013; UNDESAPD, 2015; WHO, 2019). Nearly every country in the world is experiencing growth in the number and proportion of older adults in their population. The World Health Organization (WHO) estimates that the number of people aged 65 or older will grow from an estimated 524 million in 2010 to nearly 1.5 billion by 2050 (WHO, 2011). As a result of differences in sociodemographic characteristics, the WHO (2002) sets 60 years or older as the benchmark for defining old age in low‐ and middle‐income countries and 65 years and over for populations in high‐income countries.

Although life expectancy in Africa is relatively low, projections are that the proportion of older adults in the continent will increase from 8%–19% by the year 2050 (United Nations, 2002). The population of older adults in Ghana (7%) is also expected to increase in the coming decades as a result of increasing life expectancy in the country (Ghana Statistical Service, 2013; Kwankye, 2013). Similar to many countries in Sub‐Saharan Africa, Ghana does not have social support and aged care systems for older adults (WHO, 2017). Most older adults in Ghana depend on traditional extended family structures for social protection and care (Kwankye, 2013; WHO, 2017). Evidence shows that the extended family support system in Ghana has dwindled over the years due to rural‐urban migration of the youth in search for employment opportunities in the big cities (Kpessa‐Whyte, 2018; Tawiah, 2011). Ghana as a low‐middle‐income country has a huge healthcare infrastructural deficit. In 2018, Ghana had a total hospital bed capacity of 23,829 beds for a population of nearly 30 million people (United Nations Department of Economic and Social Affairs Population Division, 2019). This implies that older adults in Ghana who need medical attention must compete with the general population for the already overburdened healthcare infrastructure. This situation could have implications on the quality of care that older adults receive in Ghanaian hospitals. An assessment of hospitalized older adults’ levels of satisfaction with the quality of nursing care will serve as an evaluation of the ability of the healthcare system in Ghana to provide safe and quality care that meets the multifaceted healthcare needs of older adults in the country. Findings of the study will also serve as an impetus for capacity building and sensitization workshops aimed to improve geriatric care.

## 2 BACKGROUND

Access to safe and quality nursing care is a basic human right and is important for the well‐being of hospitalized older adults. The “Code of Professional Conduct” for nurses in Ghana requires nurses to render quality nursing care and to maintain the dignity of all patients regardless of age, gender, race or economic status (Nursing & Midwifery Council of Ghana, 2017). Available evidence suggests that hospitalized older adults are susceptible to physical and emotional abuse (Filipska et al., 2020; Naderi et al., 2019) as a result of the decline in physical and cognitive function associated with ageing (Animasahun & Chapman, 2017; Yatsuya et al., 2017). Hospitalization also poses a threat to the physical and psychosocial well‐being of older adults (Hillman et al., 2013; Stewart & Arora, 2018). According to Hillman et al. (2013), older adults experience a myriad of negative emotions such as feeling of loneliness and isolation during hospitalization.

Efforts have been made by researchers to explore older adults experiences with care during acute hospitalization (Asmaningrum et al., 2020; Bridges et al., 2020; Kerr et al., 2020; Tadd et al., 2011). These studies have identified incidents of negative experiences that ranged from negative attitude of healthcare professionals (HCPs) (Asmaningrum et al., 2020; Calnan et al., 2013; Tadd et al., 2011) to feeling of worthlessness and powerlessness without control of care decisions (Bridges et al., 2020; Tauber‐Gilmore et al., 2018). The majority of these studies, however, have been conducted using qualitative designs with high levels of subjectivity and limitations for generalizability.

Ghana has a low literate population of older adults as the majority of its older adult population are without formal education (Alhassan & Neysmith, 2013). Older adults in Ghana often face numerous challenges when accessing healthcare services including communication difficulties with HCPs, negative attitudes of HCPs, poorly designed healthcare infrastructure and difficulties in meeting healthcare cost due to inadequate health insurance coverage (Agyemang‐Duah et al., 2019). Further, there have been media reports of older adults being refused access to healthcare services in Ghana because of lack of hospital beds for admissions (Akosa, 2018; Asamoah, 2018). The effects of these experiences on older adults’ levels of satisfaction with the quality of nursing care have not been quantitatively explored.

Efforts have also been made by researchers to quantitatively estimate inpatients’ satisfaction with nursing care to serve as an impetus for improvement in care (Ahmadabad et al., 2016; Alasad et al., 2015; Al‐Awamreh & Suliman, 2019; Chumbler et al., 2016; Haile Eyasu et al., 2016; Karaca & Durna, 2019; Özlü & Uzun, 2015; Razia et al., 2019). These studies have revealed differences in patients’ satisfaction with nursing care across different geographic locations. For instance, while more than half (52.5%) of inpatients in Ethiopia were reported to have been satisfied with nursing care (Haile Eyasu et al., 2016), this figure is higher in Nigeria where more than three quarters (81.7%) of inpatients are reportedly highly satisfied with nursing care (Mobolaji‐Olajide et al., 2020). Several factors have been identified in the literature as influencing factors for patients’ satisfaction with nursing care including gender (Alasad et al., 2015; Chumbler et al., 2016; Haile Eyasu et al., 2016; Razia et al., 2019), age, (Chumbler et al., 2016; Özlü & Uzun, 2015; Razia et al., 2019), individualized care, good nurse–patient relationship and educational attainment (Chumbler et al., 2016; Mobolaji‐Olajide et al., 2020).

Previous studies on patients’ satisfaction with nursing care in the African context (Grace, 2018; Haile Eyasu et al., 2016; Mobolaji‐Olajide et al., 2020; Yimer et al., 2021) lacked specificity for older adults as they involved all adult patients in their samples (young, middle‐aged and older adults). No study has been identified that assessed hospitalized older adults’ satisfaction with nursing care from the African perspective. Findings of studies on hospitalized older adults’ satisfaction with nursing care from other continents may not be a representation of the perspectives of hospitalized older adults in Africa due to differences in sociodemographic characteristics. This study, therefore, sought to assess the level of satisfaction with quality nursing care among older adults receiving acute healthcare services in public hospitals in the Tamale metropolis, Ghana. Nursing care is an inseparable component of health care and assessing hospitalized older adults’ level of satisfaction with the quality of nursing care constitutes an integral part of the evaluation of the healthcare system. The findings of the study will create awareness of the current quality of nursing care in the Tamale metropolis.

## 3 MATERIALS AND METHODS

### 3.1 Research design

We employed a quantitative descriptive cross‐sectional survey design in the present study. With the quantitative descriptive cross‐sectional design, measurements of both the independent and dependent variables are made on a single occasion.

### 3.2 Research setting

The study was conducted in the Tamale Metropolis which is the capital town of the Northern Region of Ghana. Tamale is Ghana's third‐largest city with a population of 360,579 people, according to the world population review (World Population Review, 2019). The town is located 600 km north of Accra, the capital city of Ghana. Study participants were recruited from the three government hospitals in the Tamale metropolis: The Tamale Teaching Hospital (TTH), the Tamale Central Hospital (TCH) and the Tamale West Hospital (TWH).

### 3.3 Population and sampling

The population for this study comprised all patients aged 60 years or more who had been on admission at any of the participating hospitals for at least three days. The total population was 421 patients. The participants were selected using a combination of quota sampling and convenience sampling methods. Quota sampling method was used to determine proportion of participants to include from each hospital; then, the participants from each hospital were selected using convenience sampling. The patients were selected based on the inclusion criteria of being 60 years or older, being admitted to the hospital for a period of three days or more, and ability to communicate effectively in the English language or Dagbani (a local dialect). Patients who were unstable and those who were not in a position to give accurate responses were excluded from the study.

### 3.4 Sample size

The sample size was determined using Yamane's formular (Yamane, 1967). This formular is used to estimate the sample size using the total population and the margin of error for a given study. The formular is expressed as follows:

$$n=N/1+N{\left(e\right)}^{2}$$

Where *n* is the sample size, *N* is the population size, and *e* is the margin of error. Given a total population of 421 patients with 0.05 margin of error, the sample size was calculated as follows:

$$n=421/1+421{\left(0.05\right)}^{2}=205.60$$

Therefore, the sample size was 206 patients.

### 3.5 Data collection tools

We gathered data for the study using a structured questionnaire. The questionnaire comprised a demographic information section and the Patient Satisfaction with Nursing Care Quality Questionnaire (PSNCQQ), developed by Laschinger et al. (2005) The PSNCQQ is a 19 item scale with three additional questions designed to tap satisfaction with the overall quality of care during the hospital admission, overall quality of nursing care and intention to recommend the hospital to family and friends. Each item is measured on a 5‐point Likert scale from strongly agree to strongly disagree. The PSNQQ was a suitable scale for the study as it was designed to solicit feedback on the quality of nursing care from patients’ perspectives. The questionnaire has an excellent internal consistency with a Cronbach's alpha value of 0.97 (Laschinger et al., 2005).

A multi‐step forward‐backward translation of the questionnaire from the English language to Dagbani and back to the English language was undertaken by two professionals who are prolific speakers of both languages. The two professionals independently translated the original questionnaire from the English language to Dagbani. Following the initial translation, they met to discuss the translation process and produced a single copy of the translated questionnaire by consensus. A third professional who is a native speaker of Dagbani and also proficient with the English language was contacted to translate the Dagbani version of the questionnaire back to the English language.

Content validity of the questionnaire was assessed with five experienced nurses using the Content Validity Index (CVI) (Polit & Beck, 2006). The nurses were tasked to rate each item on the questionnaire and the entire questionnaire as a true measure of quality nursing care on a 4‐point Likert scale (1‐Not relevant; 2‐Somewhat relevant; 3‐Quite relevant; and 4‐Highly relevant). Acceptable CVI scores of 0.8 and 0.74 for the scale‐CVI and item‐CVI were recorded respectively. Face validity of the questionnaire was assessed through a focus group discussion with five hospitalized older patients. The patients were asked about their opinion on each item on the questionnaire as a true measure of quality nursing care. The discussion was focussed on linguistical and semantical aspect of the questionnaire without a change to the content. Nearly, all the patients identified the items of the questionnaire as a true measure of quality nursing care.

### 3.6 Data collection procedure

Following ethics and institutional approval, we visited the wards of the participating hospitals and identified patients who met the inclusion criteria. We sought permission from the ward nurses and approached the identified patients to explain the study to them, what participation involved and participant rights. We encouraged them to ask questions about the study and assured them that participation was totally voluntary. Those who agreed to participate in the study were recruited after signing an informed consent. We personally administered the questionnaires to all participants. For patients who were not literate, we read out the questionnaire to them in the local dialect and recorded their responses. Participants were allowed to complete the questionnaire at their own convenience. Data collection took place over a period of four week (from 18 March 2019 to 14 April 2019).

### 3.7 Data analysis

We analysed the data using the Statistical Package for Social Sciences (SPSS) version 22 (IBM, 2015). We used descriptive statistics to summarize demographic and other background characteristics of participants and to present their levels of satisfaction with quality of nursing care. We determined patients’ levels of satisfaction with the quality of nursing care by calculating the average total scores of all items on the PSNCQQ. This calculation resulted in a range of scores from 22–110. We put the patients into three levels of satisfaction based on their total scores on the PSNCQQ. The levels were low satisfaction (22–50), moderate satisfaction (51–80) and high satisfaction (81–110). We also used the independent sample *t* test to assess differences in the level of satisfaction with quality of nursing care between males and females. Lastly, we conducted a one‐way ANOVA to determine differences in the level of satisfaction with quality of nursing care based on religion, level of education and the admitting hospitals.

### 3.8 Ethical considerations

We obtained ethical clearance for the study. We also sought permission from the management of the participating hospitals before the data collection process began. Besides, we explained the purpose and potential benefits and risks for participating in the study to the participants in the English language or Dagbani (local dialect) before the data collection. The participants were given a 24 hr period to consider their participation from the time they were approached to take part in the study. Participants who agreed to take part in the study were made to give their consent by signing the consent form. We also informed the participants that they could withdraw from the study at any time even after giving their consent and that refusal to participate in the study will not affect the quality of service they received from the hospitals.

## 4 RESULTS

### 4.1 Demographic/background characteristics of participants

Out of the total number of participants whom the questionnaires were administered to, 206 participants eventually completed the survey. The majority of the participants 128 (62.1%) were males, without formal education (54.9%), married 145 (70.4%) and belonging to the Islamic faith 160 (77.7%). Other characteristics of the participants are presented in Table 1.

**TABLE 1:** Demographic/Background Characteristics of Participants

Variables	Frequency	Percentage (%)
Gender		
Male	128	62.1
Female	78	37.9
Age		
60–65	60	29.1
66–70	49	23.8
71–75	43	20.9
76–80	33	16.0
81–85	14	6.8
>85	07	3.4
Marital status		
Single	2	1.0
Married	145	70.4
Divorced	14	6.8
Widowed	42	20.4
Others	3	1.5
Religion		
Christianity	42	20.4
Islam	160	77.7
Others	4	1.9
Level of education		
No formal education	113	54.9
Basic level	16	7.8
Senior high	30	14.6
Diploma	29	14.1
Bachelor's degree	15	7.3
Master's degree	2	1.0
Ph.D.	1	0.5
Admitting facility		
Tamale Teaching Hospital	128	62.1
Tamale Central Hospital	53	25.7
Tamale West Hospital	25	12.2

### 4.2 Satisfaction with quality nursing care

A large majority of the participants 149 (72.3%) reported moderate levels of satisfaction with the quality of nursing care received at the various hospitals. With satisfaction scores ranging from 22–110, the mean score was 74.126, with a standard deviation of 9.999. Participants’ levels of satisfaction with the quality of nursing care are presented in Table 2.

**TABLE 2:** Participants’ Level of Satisfaction with Quality Nursing Care

Level of satisfaction	Frequency	Percentage (%)
Low level	1	0.5
Moderate level	149	72.3
High level	49	23.8

Note

Seven participants had incomplete responses to the scale, and their responses were excluded from the analysis.

We also compared the level of participant satisfaction with the quality of nursing care across the three different hospitals. The findings are presented in Table 3.

**TABLE 3:** Patients’ Satisfaction with Quality of Nursing Care in the Participating Hospitals

Hospital	Frequency	Mean satisfaction score	Standard deviation
Tamale Teaching Hospital	140	70.235	9.272
Tamale Central Hospital	50	76.078	8.095
Tamale West Hospital	15	73.473	9.088

### 4.3 Association between satisfaction with nursing care and selected background characteristics of participants

To determine factors influencing satisfaction with nursing care, we compared the level of satisfaction of participants using different demographic characteristics of the participants. To assess for differences in the level of satisfaction between males and females, the independent sample *t* test was used. The test revealed that the difference in satisfaction scores between males and females was not statistically significant (*t*(197) = −0.918, *p* = .360). A one‐way ANOVA was also conducted to assess for differences in the level of satisfaction based on participants’ religion, level of education and admitting facility. The results showed that there were no statistically significant differences in the level of satisfaction of participants based on their religion (*F*(2, 196) = 0.139, *p* = .870) and level of education (*F*(6, 192) = 0.848, *p* = .535). However, the admitting facilities accounted for statistically significant differences in the level of satisfaction of participants (*F*(6,192) = 8.033, *p* = .001). A Tukey post hoc test revealed that the mean satisfaction score for participants admitted to the Tamale Central Hospital was statistically significantly higher (76.078 ± 8.095, *p* = .001) than those admitted to Tamale Teaching Hospital (70.235 ± 9.272, *p* = .001). The satisfaction scores for participants admitted to the Tamale West Hospital were not statistically different from the other facilities (*p* = .586). Further details of the one‐way ANOVA are presented in Table 4.

**TABLE 4:** One‐way ANOVA for Satisfaction Scores Based on Religion, Level of Education and Admitting Facility

Parameters	Sum of squares	Df	Mean square	F	Sig.
Religion					
Between groups	27.547	2	13.774	0.139	0.870
Within groups	19,376.312	196	98.859		
Level of education					
Between groups	500.765	6	83.461	0.848	0.535
Within groups	18,903.094	192	98.454		
Admitting facility					
Between groups	1297.875	2			
Within groups	16,319.093	196	648.93	8.033	0.001

## 5 DISCUSSION

The findings of our study indicated that a greater percentage of the participants were moderately satisfied with the quality of nursing care at the various government hospitals in the Tamale metropolis. Only a smaller percentage of the participants reported high levels of satisfaction with the quality of nursing care. In tandem with our findings, previous studies have reported that the majority of hospitalized older adults are either dissatisfied or moderately satisfied with the quality of care rendered at hospitals (Ahmadabad et al., 2016; Shady et al., 2018). Nearly three quarters of the participants were unsatisfied with the quality of nursing care in Shady et al.’s (2018) study, while the same proportion of participants reported moderate levels of satisfaction with care in a study by Ahmadabad et al. (2016). Only a small fraction of our participants reported high levels of satisfaction with the nursing care, and this came as no surprise to the research team. Ghana does not have a geriatric nursing programme or an aged care support system for its older adult population. As a consequence, older adults in Ghana compete with the general population for healthcare services that is already overstretched due to inadequate infrastructure. As part of measures to improve care for older adults, hospital managements and other relevant stakeholders should organize capacity building and sensitization workshops on the rudiments of gerontological nursing in the interim. In the long run, the government must improve on the healthcare infrastructure in Ghana and introduce specialist geriatric nursing programmes that will equip nurses with the requisite skills to meet the multifaceted needs of hospitalized older adults.

Regarding factors influencing satisfaction with the quality of nursing care, we found that gender, religion and educational attainment did not appear to influence participants’ level of satisfaction with nursing care. In conformity with our findings, several previous studies have reported no statistically significant difference in the levels of satisfaction with the quality of nursing care between older male and female patients (Kazerooni et al., 2019; Özlü & Uzun, 2015). Other studies, on the contrary, have observed a statistically significant difference in the levels of satisfaction with nursing care between male and female older adults, with females more likely to be satisfied with the quality of nursing care than their male counterparts (Chumbler et al., 2016; Haile Eyasu et al., 2016). Contrary to our findings, several studies in the past have identified differences in hospitalized older adults’ levels of satisfaction with nursing care based on educational attainments (Chumbler et al., 2016; Dehghani Ahmadabad et al., 2016; Kazerooni et al., 2019). Chumbler et al. (2016) reported an inverse relationship between higher educational attainment and satisfaction with the quality of nursing care. Older adults with high school and college education in their study scored low levels of satisfaction with nursing care as compared with those with less than 8th‐grade education. Kazerooni et al. (2019) and Dehghani Ahmadabad et al. (2016) assert in their studies that older adults with higher education pay more attention to different factors in satisfaction and hence may be less satisfied as compared to older adults without formal education. Differences in demographic characteristics of participants between our study and that of previous studies (Chumbler et al., 2016; Dehghani Ahmadabad et al., 2016; Kazerooni et al., 2019) might have accounted for the differences in findings. For instance, while the majority of our participants (54.9%) had no formal education, 94.6% of the patients in Chumbler et al.'s. (2016) study had high school education or higher.

However, we did find a statistically significant differences in participants’ level of satisfaction with the quality of nursing care based on the hospitals where they were admitted. This finding is incongruent with the findings of a similar study conducted in Makkah Al Mukramah, where there was no statistically significant difference in participants’ levels of satisfaction with quality nursing care among the three different hospitals involved in the study (El‐Nagger et al., 2012). It was, however, surprising to us that participants at the Tamale Central Hospital reported higher levels of satisfaction with the quality of nursing care than those at the Tamale Teaching Hospital. This is because the Tamale Teaching Hospital is a tertiary health facility while the Tamale Central Hospital is a secondary health facility.

## 6 CONCLUSION AND RECOMMENDATIONS

Our findings revealed that the majority of older adults in the Tamale Metropolis are moderately satisfied with the quality of nursing care they receive at the various public hospitals in the metropolis with just a small proportion reporting high levels of satisfaction with nursing care. We also observed that hospitalized older adults at the Tamale Central Hospital are more satisfied with the quality of nursing care that those admitted at the Tamale Teaching Hospital. We conclude that besides the differences in the admitting facilities, other demographic characteristics such as gender and religion do not influence hospitalized older adults' levels of satisfaction with quality nursing care. There is a need for capacity building and sensitization workshops on the rudiments of geriatric care for nurses in the metropolis to enable them to improve on the quality of nursing care services for hospitalized older adults. The government of Ghana must introduce a specialist programme in geriatric nursing to equip nurses with the requisite knowledge and skill that will meet the multifaceted needs of hospitalized older adults.

## CONFLICTS OF INTEREST

The authors declare no conflict of interest.

## AUTHOR CONTRIBUTIONS

The first author conceptualized the research topic and drafted the proposal for the project. He also wrote the discussion, conclusion and recommendations of the paper with support from the other authors. The second and fourth authors were responsible for the collection of the data for the project, while the third author assisted in the analysis of the data and writing of the report of the findings.

## Data Availability

The data sets generated and analysed during the current study are available from the corresponding author on request.

## References

[nop21169-bib-0001] Agyemang‐Duah, W. , Peprah, C. , & Peprah, P. (2019). Barriers to formal healthcare utilisation among poor older people under the livelihood empowerment against poverty programme in the atwima nwabiagya district of Ghana. BMC Public Health, 19(1), 1185. 10.1186/s12889-019-7437-2 31462254 PMC6714403

[nop21169-bib-0002] Ahmadabad, A. D. , Bahrevar, V. , & Zeinali, A. (2016). Elderly Patients’ satisfaction with provided services in yazd shahid sadoughi hospital. Elderly Health Journal, 2(1), 45–49.

[nop21169-bib-0003] Ailshire, J. , & Crimmins, E. (2011). Psychosocial factors associated with longevity in the United States: Age differences between the old and oldest‐old in the health and retirement Study. Journal of Aging Research, 2011, 1–10. 10.4061/2011/530534 PMC3199053 22028969

[nop21169-bib-0004] Akosa, A. B. (2018). ‘No bed’ syndrome, a telling phenomenon of Ghana’s health care. Graphic Online. https://www.graphic.com.gh/features/opinion/no‐bed‐syndrome‐a‐telling‐phenomenon‐of‐ghana‐s‐health‐care.html

[nop21169-bib-0005] Alasad, J. , Abu Tabar, N. , & AbuRuz, M. E. (2015). Patient satisfaction with nursing care: Measuring outcomes in an international setting. JONA: The Journal of Nursing Administration, 45(11), 563–568. 10.1097/NNA.0000000000000264 26492148

[nop21169-bib-0006] Al‐Awamreh, K. , & Suliman, M. (2019). Patients' satisfaction with the quality of nursing care in thalassemia units. Applied Nursing Research, 47, 46–51. 10.1016/j.apnr.2019.05.007 31113547

[nop21169-bib-0007] Alhassan, I. P. , & Neysmith, S. (2013). Policy implications of population ageing in West Africa. International Journal of Sociology and Social Policy, 33(3/4), 186–202. 10.1108/01443331311308230

[nop21169-bib-0008] Animasahun, V. J. , & Chapman, H. J. (2017). Psychosocial health challenges of the elderly in Nigeria: A narrative review. African Health Sciences, 17(2), 575. 10.4314/ahs.v17i2.35 29062356 PMC5642086

[nop21169-bib-0009] Asamoah, K. L. (2018). No bed syndrome must stop—GHS | Ghana news agency (GNA). http://www.ghananewsagency.org/health/no‐bed‐syndrome‐must‐stop‐ghs‐134013

[nop21169-bib-0010] Asmaningrum, N. , Kurniawati, D. , & Tsai, Y. F. (2020). Threats to patient dignity in clinical care settings: A qualitative comparison of Indonesian nurses and patients. Journal of Clinical Nursing, 29(5–6), 899–908. 10.1111/jocn.15144 31855306

[nop21169-bib-0011] Bridges, J. , Collins, P. , Flatley, M. , Hope, J. , & Young, A. (2020). Older people’s experiences in acute care settings: Systematic review and synthesis of qualitative studies. International Journal of Nursing Studies, 102, 103469. 10.1016/j.ijnurstu.2019.103469 31862528

[nop21169-bib-0012] Calnan, M. , Tadd, W. , Calnan, S. , Hillman, A. , Read, S. , & Bayer, A. (2013). “I often worry about the older person being in that system”: Exploring the key influences on the provision of dignified care for older people in acute hospitals. Ageing and Society, 33(3), 465–485. 10.1017/S0144686X12000025

[nop21169-bib-0013] Cho, J. , Martin, P. , & Poon, L. (2013). Age group differences in positive and negative affect among oldest‐old adults: Findings from the georgia centenarian study. The International Journal of Aging and Human Development, 77(4), 261–288. 10.2190/AG.77.4.a 24547613

[nop21169-bib-0014] Chumbler, N. R. , Otani, K. , Desai, S. P. , Herrmann, P. A. , & Kurz, R. S. (2016). Hospitalized older adults’ patient satisfaction: Inpatient care experiences. SAGE Open, 6(2), 215824401664563. 10.1177/2158244016645639

[nop21169-bib-0015] Dehghani Ahmadabad, A. , Bahrevar, V. , & Zeinali, A. (2016). Elderly patients’ satisfaction with provided services in yazd shahid sadoughi hospital. Elderly Health Journal, 2(1), 45–49.

[nop21169-bib-0016] El‐Nagger, N. , Ahmed, S. , Elsayed, L. , & Khamis, H. (2012). Patients’ satisfaction regarding nursing care provided in different hospitals in Makkah Al Mukramah. Life Science Journal, 10(2), 421–429.

[nop21169-bib-0017] Filipska, K. , Biercewicz, M. , Wiśniewski, A. , Kędziora‐Kornatowska, K. , & Ślusarz, R. (2020). Prevalence and associated factors of elder psychological abuse‐ a cross‐ sectional screening study, based on a hospitalized community from Poland. Archives of Gerontology and Geriatrics, 90, 104152. 10.1016/j.archger.2020.104152 32623311

[nop21169-bib-0018] Ghana Statistical Service (2013). 2010 Population and housing census: The elderly in Ghana. Ghana Statistical Service.

[nop21169-bib-0019] Grace, G. (2018). Evaluation of patient satisfaction with nursing care at two public hospitals in kenya: An interventional study. Nursing & Healthcare International Journal, 2(4), 1–8. 10.23880/NHIJ-16000151

[nop21169-bib-0020] Haile Eyasu, K. , Adane, A. A. , Amdie, F. Z. , Getahun, T. B. , & Biwota, M. A. (2016). Adult patients’ satisfaction with inpatient nursing care and associated factors in an ethiopian referral hospital, Northeast, Ethiopia. Advances in Nursing, 2016, 1–7. 10.1155/2016/6308617

[nop21169-bib-0021] Hillman, A. , Tadd, W. , Calnan, S. , Calnan, M. , Bayer, A. , & Read, S. (2013). Risk, governance and the experience of care. Sociology of Health & Illness, 35(6), 939–955. 10.1111/1467-9566.12017 23356787 PMC3813989

[nop21169-bib-0022] IBM (2015). Statistical package for social sciences, (Version 21) [Windows]. IBM.

[nop21169-bib-0023] Karaca, A. , & Durna, Z. (2019). Patient satisfaction with the quality of nursing care. Nursing Open, 6(2), 535–545. 10.1002/nop2.237 30918704 PMC6419107

[nop21169-bib-0024] Kazerooni, K. , Pazokian, M. , Nasiri, M. , & Borhani, F. (2019). Expectations and satisfaction of elderly people with health services provided at a public nursing home in Iran. Revista Latinoamericana De Hipertensión, 14, 7.

[nop21169-bib-0025] Kerr, D. , Crone, R. , & Dunning, T. (2020). Perspectives about dignity during acute care for older people and their relatives: A qualitative study. Journal of Clinical Nursing, 29(21–22), 4116–4127. 10.1111/jocn.15438 32757417

[nop21169-bib-0026] Kpessa‐Whyte, M. (2018). Aging and demographic transition in Ghana: State of the elderly and emerging issues. The Gerontologist, 58(3), 403–408. 10.1093/geront/gnx205 29381779

[nop21169-bib-0027] Kwankye, S. (2013). Growing old in Ghana: Health and economic implications. Postgraduate Medical Journal of Ghana, 2(2), 88–97. https://gcps.edu.gh/wp‐content/uploads/2014/09/Growing‐old‐in‐Ghana‐health‐and‐economic‐implications.pdf

[nop21169-bib-0028] Laschinger, S. , Hall, M. , Pedersen, C. , & Almost, J. (2005). A psychometric analysis of the patient satisfaction with nursing care quality questionnaire. Journal of Nursing Care Quality, 20(3), 220–230. 10.1097/00001786-200507000-00006 15965386

[nop21169-bib-0029] Mobolaji‐Olajide, O. M. , Adereti, S. C. , Odutayo, P. O. , & Adejumo, P. O. (2020). In‐patient satisfaction with nursing care: Outcome measurement in a tertiary health facility in Lagos, Nigeria. International Journal of Africa Nursing Sciences, 13, 100264. 10.1016/j.ijans.2020.100264.

[nop21169-bib-0030] Naderi, Z. , Gholamzadeh, S. , Zarshenas, L. , & Ebadi, A. (2019). Hospitalized elder abuse in Iran: A qualitative study. BMC Geriatrics, 19(1), 307. 10.1186/s12877-019-1331-8 31718591 PMC6852990

[nop21169-bib-0031] Nursing and Midwifery Council of Ghana (2017). CODE OF PROFESSIONAL CONDUCT ‐ NURSING AND MIDWIFERY COUNCIL OF Ghana. Nursing In Ghana. https://nursinginghana.com/code‐professional‐conduct‐nursing‐midwifery‐council‐ghana/

[nop21169-bib-0032] Özlü, K. , & Uzun, Ö. (2015). Evaluation of satisfaction with nursing care of patients hospitalaized in surgical clinics of different hospitals. International Journal of Caring Sciences, 8(1), 19–24.

[nop21169-bib-0033] Polit, D. F. , & Beck, C. T. (2006). The content validity index: Are you sure you know what’s being reported? critique and recommendations. Research in Nursing & Health, 29(5), 489–497. 10.1002/nur.20147 16977646

[nop21169-bib-0034] Razia, M. S. , Nesa, M. , & Park, J. S. (2019). Patient satisfaction with nursing care in a tertiary hospital in bangladesh. Origional Research Article, 2, 5.

[nop21169-bib-0035] Shady, A. B. , Mohammed, W. G. , & Meawad, E. B. (2018). Elderly cancer patients satisfaction with quality of nursing care in day care unit at oncology center Mansoura University. IOSR Journal of Nursing a Nd Health Science (IOSR‐JNHS), 7(6), 10. 10.9790/1959-0706070110

[nop21169-bib-0036] Stewart, N. H. , & Arora, V. M. (2018). Sleep in hospitalized older adults. Sleep Medicine Clinics, 13(1), 127–135. 10.1016/j.jsmc.2017.09.012 29412979 PMC5880551

[nop21169-bib-0037] Tadd, W. , Hillman, A. , Calnan, S. , Calnan, M. , Bayer, T. , & Read, S. (2011). Right place ‐ wrong person: Dignity in the acute care of older people. Quality in Ageing and Older Adults, 12(1), 33–43. 10.5042/qiaoa.2011.0143

[nop21169-bib-0038] Tauber‐Gilmore, M. , Addis, G. , Zahran, Z. , Black, S. , Baillie, L. , Procter, S. , & Norton, C. (2018). The views of older people and health professionals about dignity in acute hospital care. Journal of Clinical Nursing, 27(1–2), 223–234. 10.1111/jocn.13877 28514523

[nop21169-bib-0039] Tawiah, E. O. (2011). Population ageing in Ghana: A profile and emerging issues. African Population Studies, 25(2), 623–645. 10.11564/25-2-249

[nop21169-bib-0040] UNDESAPD (2015). World Population Ageing 2015. United Nations.

[nop21169-bib-0041] United Nations (2002). Political declaraation and madrid international plan of action on ageing: Second world assembly on ageing. United Nations.

[nop21169-bib-0042] United Nations Department of Economic and Social Affairs Population Division . (2019). World population prospects 2019: Highlights (ST/ESA/SER.A/423), 1. Retrieved from: https://population.un.org/wpp/Publications/Files/WPP2019_Highlights.pdf

[nop21169-bib-0043] WHO . (2002). WHO | Proposed working definition of an older person in Africa for the MDS Project. WHO. http://www.who.int/healthinfo/survey/ageingdefnolder/en/

[nop21169-bib-0044] WHO (2011). Global health and aging. World Health Organization. https://www.who.int/ageing/publications/global_health.pdf

[nop21169-bib-0045] WHO (2017). Towards long‐term care systems in Sub‐Saharan Africa. World Health Organization. https://www.who.int/ageing/long‐term‐care/WHO‐LTC‐series‐subsaharan‐africa.pdf?ua=1

[nop21169-bib-0046] WHO (2019). Global health observatory data: Life expectancy. World Health Organization. https://www.who.int/gho/mortality_burden_disease/life_tables/situation_trends_text/en/

[nop21169-bib-0047] World Population Review . (2019). Population of cities in Ghana (2019). http://worldpopulationreview.com/countries/ghana‐population/cities/

[nop21169-bib-0048] Yamane, T. (1967). Statistics: An introductory analysis. 2nd. ed. Harper and Row.

[nop21169-bib-0049] Yatsuya, H. , Matsunaga, M. , Li, Y. , & Ota, A. (2017). Risk factor of cardiovascular disease among older individuals. Journal of Atherosclerosis and Thrombosis, 24(3), 258–261. 10.5551/jat.Ed064 27784850 PMC5383542

[nop21169-bib-0050] Yimer, T. N. , Mekonnen Assefa, Z. , & Gizeyatu Zengye, A. (2021). Patient satisfaction and associated factors among adults attending ART clinic at dessie refferal hospital, Amhara Region, Ethiopia. International Journal of Africa Nursing Sciences, 14, 100297. 10.1016/j.ijans.2021.100297

